# Computational Protocol to Understand P450 Mechanisms and Design of Efficient and Selective Biocatalysts

**DOI:** 10.3389/fchem.2018.00663

**Published:** 2019-01-11

**Authors:** Kersti Caddell Haatveit, Marc Garcia-Borràs, Kendall N. Houk

**Affiliations:** Department of Chemistry and Biochemistry, University of California, Los Angeles, Los Angeles, CA, United States

**Keywords:** density functional theory (DFT), theozymes, MD simulations, biocatalysis, cytochrome P450s, oxidations

## Abstract

Cytochrome P450 enzymes have gained significant interest as selective oxidants in late-stage chemical synthesis. Their broad substrate scope enables them to be good candidates for their use in non-natural reactivity. Directed evolution evolves new enzyme biocatalysts that promote alternative reactivity for chemical synthesis. While directed evolution has proven useful in developing biocatalysts for specific purposes, this process is very time and labor intensive, and therefore not easily repurposed. Computational analysis of these P450 enzymes provides great insights into the broad substrate scope, the variety of reactions catalyzed, the binding specificity and the study of novel biosynthetic reaction mechanisms. By discovering new P450s and studying their reactivities, we uncover new insights into how this reactivity can be harnessed. We discuss a standard protocol using both DFT calculations and MD simulations to study a variety of cytochrome P450 enzymes. The approach entails theozyme models to study the mechanism and transition states via DFT calculations and subsequent MD simulations to understand the conformational poses and binding mechanisms within the enzyme. We discuss a few examples done in collaboration with the Tang and Sherman/Montgomery groups toward elucidating enzyme mechanisms and rationally designing new enzyme mutants as tools for selective C–H functionalization methods.

## Introduction

Cytochrome P450s are a highly-conserved class of enzymes that contain an Fe-heme cofactor with an axial cysteine ligand that can perform different oxidation reactions on a large variety of native substrates. (Ortiz de Montellano, 2005) P450 mechanisms have been extensively studied (Shaik et al., 2010) and require the oxidative generation of the reactive cofactor species, an iron(IV)-oxo (Fe^IV^ = O) radical cation. With this reactive species, the oxidation step in the hydroxylation reaction occurs via a hydrogen abstraction step followed by a radical rebound step between the cofactor and substrate (Meunier et al., 2004). Due to their broad substrate scope (Durairaj et al., 2016) and the growing interest developing selective methods for late-stage C–H oxidations, P450s have been utilized extensively as biocatalysts for many applications (Kumar, 2010), particularly those that are difficult to perform with chemocatalysts. Cytochrome P450s are an excellent starting point for developing biocatalysts, and many great successes (Loskot et al., 2017) in modifying and engineering P450s have come from experimental directed evolution (non-rational) approaches (Romero and Arnold, 2009), pioneered by Nobel Laureate, Frances Arnold. However, these require many rounds of screening to arrive at a biocatalyst tailored for a specific purpose. This process can be sped up by developing a better understanding about how these P450s function, particularly by using computational methods. Rational approaches utilizing computational methods to study these enzymes can facilitate quicker access understanding their reactivity and predicting better variants.

**Figure 1:**
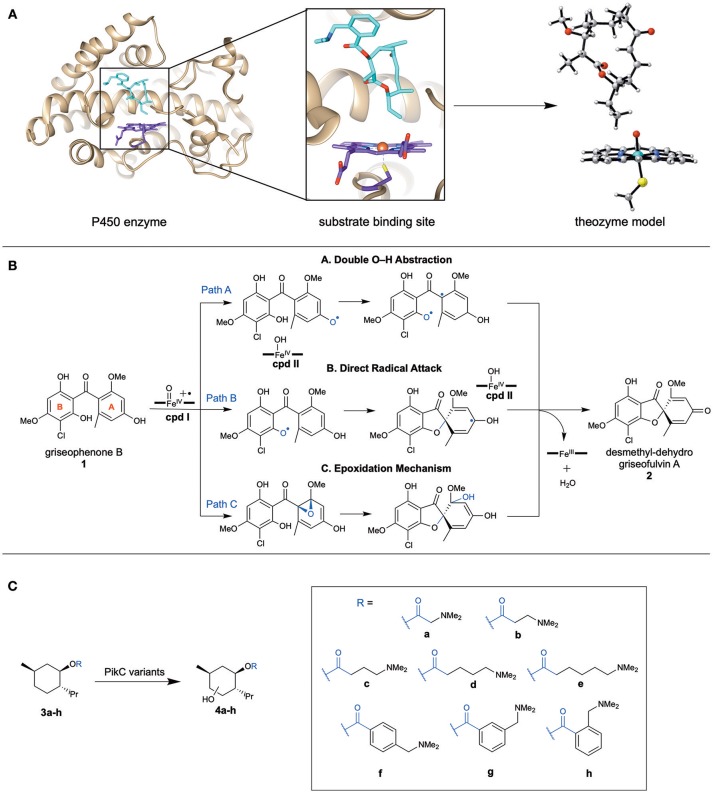
**(A)** Representative scheme displaying a chosen theozyme to study an enzyme by DFT methods. **(B)** The three mechanistic pathways proposed to be catalyzed by GsfF to generate **2**, a griseofulvin precursor. **(C)** The menthol based substrates with linkers of various lengths chosen to probe the binding mechanism with PikC variants.

While enzymes are complex systems, and their study can require elaborate computational methods, the Houk group has developed a simpler standard approach to understanding the mechanisms of complex (metallo)enzymes (particularly P450s) with great success. We utilize quantum mechanics (QM) calculations to study the transition states and mechanisms of theozyme (“theoretical enzyme” Tantillo et al., 1998) models (Figure 1A), a truncated portion of the enzyme that includes catalytically relevant active site residues and cofactors along with the substrate. We investigate the intrinsic mechanistic preferences of such transformations to understand which type of catalysis by the enzyme is most likely. Others have also utilized more rigorous approaches to study the enzyme catalysis, such as the cluster model (CM) approach used by Siegbahn, Thiel, and Himo (Siegbahn and Crabtree, 1997; Siegbahn and Himo, 2009, 2011; Blomberg et al., 2014) and QM/MM (Warshel and Levitt, 1976; Senn and Thiel, 2009; Karasulu and Thiel, 2015; Aranda et al., 2016) methods. In the beginning, CM were quite comparable to theozymes, but with the advance of computational power, CM has become more complex, and expanded to include systems of 200 atoms with other constraints on the amino acids residues to more accurately reflect the shape of the active site. QM/MM has been utilized to study the effects that mutations have on substrate binding. While these techniques are often more accurate and include the entire enzyme system, our approach is a more rapid method to study various enzyme systems (particularly P450s) and to understand their intrinsic preferences.

We combine DFT calculations with molecular dynamics (MD) simulations to explore the selectivities of enzymes and mutants. MD simulations allow us to study the substrate binding poses and positioning relative to the Fe = O active species. We can compare these geometries from MD simulations to the ideal transition state geometries determined from DFT to establish the enzyme's selectivity control. This method has allowed us to propose mutations to improve catalytic activity of enzymes. This review will focus on a few recent examples from the Houk group, first using QM theozymes to understand the feasibility of various mechanistic pathways within an enzyme, and subsequently, a combination of both QM and MD simulations to assess how the enzyme controls the reactivity and selectivity in a variety of P450 enzymes. Ultimately, we describe predictions to develop more efficient and selective enzyme mutants.

## Use of Quantum Mechanics (QM) Theozyme Models to Understand Novel Enzymatic Mechanisms

The Houk group has used quantum mechanical DFT calculations to elucidate the mechanisms of novel P450 enzymes discovered in biosynthetic pathways. These demonstrate promiscuous, yet selective, reactivity or new oxidation reactivity that has great potential for biocatalysis. By modeling the reaction using a theozyme that includes the substrate and a truncated Fe-oxo heme active species, this method leads to the intrinsically preferred mechanism. Here, we discuss an example of the mechanism for a novel C–O bond formation performed by a P450 enzyme, GsfF, to generate the natural product, griseofulvin.

In 2016, the Tang group discovered GsfF (Chooi et al., 2010; Cacho et al., 2013) from the *gsf* gene cluster responsible for the synthesis of the natural product, griseofulvin. GsfF catalyzes an oxidative cyclization that generates the spirocyclic core within the final product. Initially, two different mechanisms, a double O–H abstraction (Pathway A) and epoxidation (Pathway C), were proposed (Figure 1B). Because it was difficult to determine a plausible mechanism experimentally, the various mechanisms were studied computationally (Grandner et al., 2016). An alternative direct radical attack C–O bond formation mechanism (Pathway B) was proposed within this paper to avoid diradical formation (Figure 1B). These mechanisms were studied computationally using DFT methods [at (U)B3LYP-D3(BJ)/LanL2DZ(Fe)/6-311+G(d,p)// (U)B3LYP/LanL2DZ(Fe)/6-31G(d) levels] with a theozyme compromised of a truncated Fe porphyrin model with the substrate (Figure 1A).

Pathway C was quickly discarded as a possibility due to the high barrier (18.7 kcal/mol) as compared to the double O–H abstraction and direct radical attack mechanism that are 0.5 and 0.0 kcal/mol, respectively. The differences between pathways A and B are small (0.5 kcal/mol), and therefore further analysis was considered to distinguish the most plausible mechanism. Pathway A requires a diradical intermediate, which is highly reactive. Due to the lack of crystal structure of GsfF, homology modeling was used to evaluate the possible binding positions of the substrate in the active site. Pathway A requires the substrate to reorient its binding pose within the enzyme between the mechanistic steps, which is likely not feasible without deleterious side reactions involving the reactive diradical intermediate. Consequently, Pathway B, which doesn't require substrate reorientations, is proposed to be the most plausible pathway.

## Combination of Theozymes and Molecular Dynamics Simulations as a tool to understand P450s within Biosynthetic Pathways

While the use of QM and theozyme models provides significant information on the mechanisms of novel enzymatic catalysis of P450s, we usually combine QM and MD to obtain more knowledge of enzyme reactivity. DFT calculations of model theozymes demonstrates the intrinsic preferences for oxidation sites with P450 enzymes, while the MD simulations illustrate the various conformations and binding poses that the substrate can explore within the active site. We describe several collaborations with the Sherman and Montgomery groups to understand various P450 intrinsic reactivity for oxidations, how the enzyme controls binding orientations to overcome that innate selectivity, and how new mutations can enhance selectivity and activity.

### Prediction of Mutations to Improve WT Catalytic Activity in a P450 Monooxygenase

The Sherman group discovered a cytochrome P450 monooxygenase, PikC, a promiscuous enzyme that hydroxylates 12- and 14- membered macrolides, YC-17 and narbomycin (Xue et al., 1998). Co-crystal structures with both YC-17 and narbomycin revealed that the source of this selective, yet promiscuous, scope is due to a salt bridge interaction between the desosamine sugar anchor on the macrolides and E94 residue, enabling different substrates to react similarly if they maintain the desosamine sugar (Sherman et al., 2006). This interesting discovery led us to use PikC as the starting point for engineering a P450 biocatalyst that performs selective hydroxylations on a broad set of unnatural cyclic substrates. While the co-crystal structure provided great insights into one critical aspect of the binding mechanism, a dynamic view of PikC by utilizing both MD and QM enabled a rapid design of substrates and improved mutants (Narayan et al., 2015).

**Figure 2:**
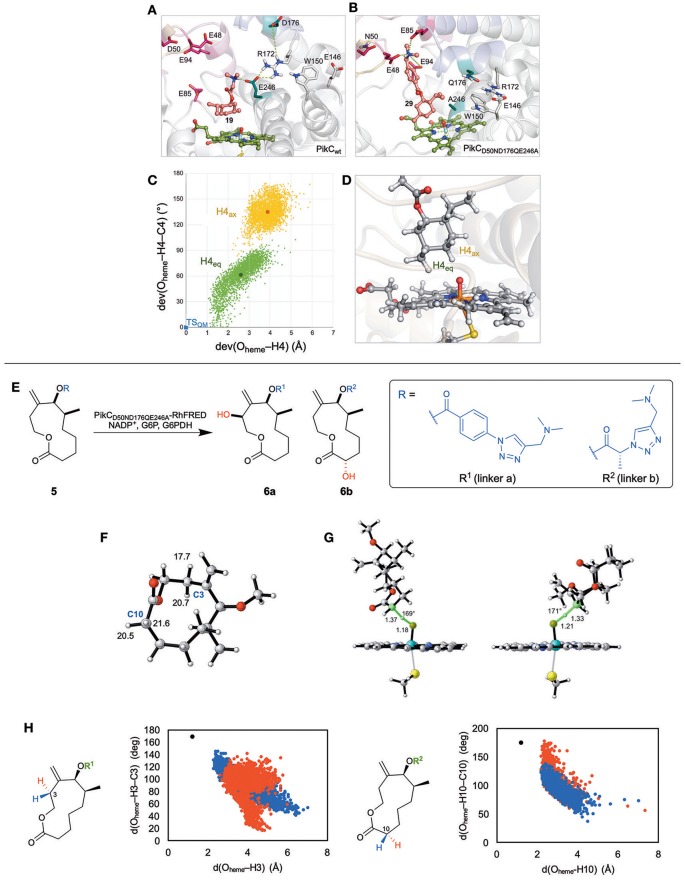
**(A)** The detrimental binding interactions of menthol-based substrate with E246 in WT PikC enzyme. **(B)** The beneficial binding interactions of menthol-based substrate with E48, E85, and E94 in triple mutant PikC, D50N/D176Q/E246A. **(C)** Orientation of H4_eq_ and H4_ax_ relative to the reactive iron-oxo species from 0.5 μs MD trajectories. Each point represents a simulation snapshot. The x-axis captures deviations of O_heme_−H distances and y-axis of O_heme_−H–C angles from DFT optimized transition state (in blue). **(D)** Representative snapshot from MD simulations showing the closer proximity of equatorial hydrogen to active Fe-oxo species. **(E)** The regio and stereoselectivity of macrolactone substrate, **5**, with different linkers **a** and **b** in PikC triple mutant. **(F)** The lowest energy conformer of truncated **5** with C–H abstraction barriers for both axial and equatorial hydrogens at C3 and C10. **(G)** The geometry of the C–H abstraction transition state for the lowest energy hydrogen (equatorial) for C3 (left) and C10 (right). **(H)** Orientation of axial (red) and equatorial (blue) hydrogens on C3 in **5a** (left) and C10 in **5b** (right) relative to the reactive iron-oxo species from 500 ns MD trajectories. Each point represents a simulation snapshot. The x-axis captures the O_heme_−H distances and y-axis of O_heme_−H–C angles compared to that of DFT optimized transition state (in black). Adapted with permission from references Narayan et al. (2015) and Gilbert et al. (2017).

Menthol was chosen as a model non-native substrate due to the variety of C–H bonds and the vast amount of information on previous C–H functionalization attempts. MD simulations were performed on several menthol-based substrates that contain various lengths of synthetic anchors (Figure 1C). From those simulations, it was discovered that when the linker is too short (**3a**), the salt bridge interactions with E94/E85 are lost, and alternatively it develops a new interaction with E246, which unproductively orients the substrate such that unreactive C–H bonds are aimed at the iron-oxo reactive site (Figure 2A). Simulations also showed that the carboxylate group in D176 can unproductively interact with the substrate, forcing it to lose its close proximity to the Fe = O active species. It was also discovered that the loss of E94/E85 interactions destabilize the tertiary structure forcing PikC to adopt its open conformation, the preferred conformation in the apo state. However, when the linkers are longer (**3e**) and contain more rigid groups such as phenyls (**3f**), salt bridge interactions with E94/E85 give a new stabilizing interaction with E48 while avoiding the harmful E246 interaction. Experimentally, a PikC D50 N mutant demonstrated improved reactivity and the MD simulations confirmed this. Furthermore, it was predicted that PikC had greater selectivity for (−) menthol over (+) menthol as the latter substrate left the active site after 100 ns of MD simulations or oscillated in and out of the pocket, depending on the synthetic anchor group. Experimental results corroborated these predictions.

From these findings, we predicted two key mutation points, E246 and D176, that would improve the efficiency of the enzymes. From the mutagenesis experiments, the triple mutant PikC_D50N/E176Q/E246A_, showed an increase in total turnover number (TTN) for all the unnatural substrates tested. The MD simulations demonstrated a higher frequency of closed conformations and persistent favorable salt bridge interactions in the triple mutant (Figure 2B) versus PikC_wt_ or single mutant PikC_D50N_.

While MD allowed us to predict essential mutations to improve catalysis in PikC, QM theozyme calculations were utilized to develop a model to predict the site selectivity of hydroxylation of these unnatural substrates. C–H abstraction transition states were computed for all the possible C–H bonds on menthol and C4 position was determined as the most reactive bond. In addition, the stereoselectivity at C4 was analyzed and a minimal difference in preference was found as the axial hydrogen abstraction barrier is lower by 0.7 kcal/mol. The chiral environment of the enzyme is responsible for biasing for one stereoisomer, thus overriding the intrinsic reactivity. Key geometric parameters, H–O distance and O–H–C angles, from snapshots of 0.5 μs MD simulations were compared to the ideal geometry from DFT calculations (Figures 2C,D). This analysis indicated that the enzyme exposes the equatorial hydrogen in the appropriate geometry to the iron-oxo species more than axial hydrogen. Experimentally, hydroxylations of these substrates gave results consistent with the computational predictions.

### Model for Origin of Selectivity for Salt Bridge Anchor Bound Substrate in a P450 Mutant

With this triple mutant PikC, the scope of the biocatalyst for oxidation of more complex substrates was explored. Anchor groups were simplified to have greater control of the selectivity, and we probed the origins of selectivity controlled by various anchors.

Our computational approach involved QM theozymes and MD simulations to predict the selectivities of substrate, **5**, modified with two linkers, **a** and **b** (Figure 2E). DFT calculations of the truncated system were utilized to analyze the intrinsic preference for oxidation for the various C–H bonds within the macrocycle (Gilbert et al., 2017). Positions C3 and C10 were determined to have the lowest C–H abstraction barriers in the two lowest energy conformers when using truncated model of macrolactone, **5**, where the linker was simplified as a methyl group (Figure 2F). These results are consistent with the two regioisomers observed experimentally. The stereochemistry at each of these positions was analyzed by comparing the C–H abstraction barrier for the axial and equatorial hydrogen at both C3 and C10. For both positions, the equatorial hydrogen abstraction had a lower energy barrier, due to better conjugation with the exocyclic olefin for C3 and less distortion to obtain conjugation for C10 (Figure 2G).

MD simulations were then performed on **5** attached with either linker **a** or linker **b**. These were docked into the crystal structure of the PikC mutant. MD simulation snapshots with linker **a** demonstrated that both C3 and C2 positions were maintained close to the reactive iron-oxo cofactor. Despite the favorable proximity of both positions, a C3–H reacts more rapidly than C2–H, due to its lower C–H abstraction barrier (5 kcal/mol lower) as shown by DFT calculations. The stereoselectivity of reaction at C3 was analyzed by comparing the geometry for C–H abstraction of both the axial and equatorial positions during MD trajectories to that of the ideal DFT transition state (Figure 2H). These results show that the PikC mutant active site bends the substrate so that the equatorial hydrogen has the appropriate orientation C–H abstraction. The same procedure was performed on the substrate with linker **b** which points the C10 close to the iron-oxo species. For C10, the axial hydrogen is in a geometry better for C–H abstraction (Figure 2H). However, the barrier for the equatorial C–H is much lower than that for the axial C–H. This process was repeated for many substrates, and experimental results were consistent with their predicted selectivity.

## Conclusion

We have described several cases where we have combined QM theozyme models and MD simulations to understand enzymatic reactivities and selectivities. This computational protocol provides understanding of enzymatic mechanisms and substrate conformational space for various cytochrome P450s. We used this knowledge to predict mutations to establish better biocatalyst variants and predict the selectivities on natural and non-natural substrates. Although we showed some successful applications of this protocol, one of its limitations is that accurate energy barriers, that can be directly compared with experimental values, cannot be obtained. For these purposes, more computationally expensive methods and protocols are required. For instance, multiple QM/MM calculations can be performed to get more accurate free energies on different conformations sampled by the enzymatic system during MD simulations. This better accounts for the catalytically relevant conformations explored by the enzyme in solution. Although the accuracy of these methods is well-established, the large amount of computational time needed dramatically limit the speed and generality of those protocols.

## Author Contributions

All authors listed have made a substantial, direct and intellectual contribution to the work, and approved it for publication.

### Conflict of Interest Statement

The authors declare that the research was conducted in the absence of any commercial or financial relationships that could be construed as a potential conflict of interest.

## References

[B1] Aranda J. Zinovjev K. Swiderek K. Roca M. Tuñón I. (2016). Unraveling the reaction mechanism of enzymatic c5-cytosine methylation of DNA. A combined molecular dynamics and QM/MM study of wild type and Gln199 variant. ACS Catal. 6, 3262–3276. 10.1021/acscatal.6b00394

[B2] Blomberg M. R. Borowski T. Himo F. Liao R.-Z. Siegbahn P. E. (2014). Quantum chemical studies of mechanisms for metalloenzymes. Chem. Rev. 114, 3601–3658. 10.1021/cr400388t 24410477

[B3] Cacho R. A. Chooi Y.-H. Zhou H. Tang Y. (2013). Complexity generation in fungal polyketide biosynthesis: a spirocycle-forming P450 in the concise pathway to the antifungal drug griseofulvin. ACS Chem. Biol. 8, 2322–2330. 10.1021/cb400541z 23978092 PMC3821396

[B4] Chooi Y.-H. Cacho R. Tang Y. (2010). Identification of the viridicatumtoxin and griseofulvin gene clusters from *Penicillium aethiopicum*. Chem. Biol. 17, 483–494. 10.1016/j.chembiol.2010.03.015 20534346 PMC2884005

[B5] Durairaj P. Hur J.-S. Yun H. (2016). Versatile biocatalysis of fungal cytochrome P450 monooxygenases. Microb. Cell Fact. 15:125. 10.1186/s12934-016-0523-6 27431996 PMC4950769

[B6] Gilbert M. M. DeMars M. D. Yang S. Grandner J. M. Wang S. Wang H.. (2017). Synthesis of diverse 11- and 12-membered macrolactones from a common linear substrate using a single biocatalyst. ACS Cent Sci. 3, 1304–1310. 10.1021/acscentsci.7b00450 29296671 PMC5746868

[B7] Grandner J. M. Cacho R. A. Tang Y. Houk K. N. (2016). Mechanism of the P450-catalyzed oxidative cyclization in the biosynthesis of griseofulvin. ACS Catal. 6, 4506–4511. 10.1021/acscatal.6b01068 28503354 PMC5425163

[B8] Karasulu B. Thiel W. (2015). Amine oxidation mediated by N-methyltryptophan oxidase: computational insights into the mechanism, role of active-site Residues, and covalent flavin binding. ACS Catal. 5, 1227–1239. 10.1021/cs501694q

[B9] Kumar S. (2010). Engineering cytochrome P450 biocatalysts for biotechnology, medicine, and bioremediation. Expert Opin. Drug Metab. Toxicol. 6, 115–131. 10.1517/17425250903431040 20064075 PMC2811222

[B10] Loskot S. A. Romney D. K. Arnold F. H. Stoltz B. M. (2017). Enantioselective total synthesis of nigelladine a via late-stage c–h oxidation enabled by an engineered P450 enzyme. J. Am. Chem. Soc. 139, 10196–10199. 10.1021/jacs.7b05196 28721734 PMC5679227

[B11] Meunier B. de Visser S. P. Shaik S. (2004). Mechanism of oxidation reactions catalyzed by cytochrome P450 enzymes. Chem. Rev. 104, 3947–3980. 10.1021/cr020443g 15352783

[B12] Narayan A. R. Jiménez-Osés G. Liu P. Negretti S. Zhao W. Gilbert M. M. (2015). Enzymatic hydroxylation of an unactivated methylene C–H bond guided by molecular dynamics simulations. Nat. Chem. 7, 653–660. 10.1038/nchem.2285 26201742 PMC4518477

[B13] Ortiz de Montellano P. (2005). Cytochrome P450: Structure, Mechanism, and Biochemistry, 3e. New York, NY: Kluwer Academic/Plenum Publishers.

[B14] Romero P. A. Arnold F. N. (2009). Exploring protein fitness landscapes by directed evolution. Nat. Rev. Mol. Cell Biol. 10, 866–876. 10.1038/nrm2805 19935669 PMC2997618

[B15] Senn H. M. Thiel W. (2009). QM/MM methods for biomolecular systems. Angew. Chem. Int. Ed. 48, 1198–1229. 10.1002/anie.200802019 19173328

[B16] Shaik S. Cohen S. Wang Y. Chen H. Kumar D. Thiel W. (2010). P450 enzymes: their structure, reactivity, and selectivity-modeled by QM/MM calculations. Chem. Rev. 110, 949–1017. 10.1021/cr900121s 19813749

[B17] Sherman D. H. Li S. Yermalitskaya L. V. Kim Y. Smith J. A. Waterman M. R.. (2006). The structural basis for substrate anchoring, active site selectivity, and product formation by P450 PikC from *Streptomyces venezuelae*. J. Biol. Chem. 281, 26289–26297. 10.1074/jbc.M605478200 16825192 PMC2939096

[B18] Siegbahn P. E. Himo F. (2009). Recent developments of the quantum chemical cluster approach for modeling enzyme reactions. J. Biol. Inorg. Chem. 14, 643–651. 10.1007/s00775-009-0511-y 19437047

[B19] Siegbahn P. E. M. Crabtree R. (1997). Mechanism of C–H activation by diiron methane monooxygenases: quantum chemical studies. J. Am. Chem. Soc. 119, 3103–3113. 10.1021/ja963939m

[B20] Siegbahn P. E. M. Himo F. (2011). The quantum chemical cluster approach for modeling enzyme reactions. Rev. Comput. Mol. Sci. 1, 323–336. 10.1002/wcms.13 19437047

[B21] Tantillo D. J. Chen J. Houk K. N. (1998). Theozymes and compuzymes: theoretical models for biological catalysis. Curr. Opin. Chem. Biol. 2, 743–750. 10.1016/S1367-5931(98)80112-9 9914196

[B22] Warshel A. Levitt M. (1976). Theoretical studies of enzymic reactions: dielectric, electrostatic and steric stabilization of the carbonium ion in the reaction of lysozyme. J. Mol. Biol. 103, 227–249. 10.1016/0022-2836(76)90311-9 985660

[B23] Xue Y. Wilson D. Zhao L. Liu H. W. Sherman D. H. (1998). Hydroxylation of macrolactones YC-17 and narbomycin is mediated by the PikC-encoded cytochrome P450 in *Streptomyces venezuelae*. Chem. Biol. 5, 661–667. 10.1016/S1074-5521(98)90293-9 9831532

